# Serological evidence of Eastern equine encephalitis circulation in equids in Pará state, Brazil

**DOI:** 10.29374/2527-2179.bjvm001720

**Published:** 2021-03-31

**Authors:** Josynélia do Socorro da Silva Sena Nunes, Lívia Medeiros Neves Casseb, Ricardo José de Paula Sousa Guimarães, Wilgner Duarte Magalhães Reis, Bruno de Cássio Veloso de Barros, Milene Silveira Ferreira, Jannifer Oliveira Chiang, Helder Henrique Costa Pinheiro, Pedro Fernando da Costa Vasconcelos, Ana Cecília Ribeiro Cruz

**Affiliations:** 1 Veterinarian, MSc., Programa de Pós-Graduação em Virologia (PPGV), Instituto Evandro Chagas (IEC), Ananindeua, PA, Brazil; 2 Veterinarian, DSc., Seção de Arbovirologia e Febres Hemorrágicas, IEC, Ananindeua, PA, Brazil; 3 Biologist. DSc., Setor de Geoprocessamento, IEC, Ananindeua, PA, Brazil; 4 Graduate Student in Biomedicine, Universidade Estadual do Pará (UEPA), Marabá, PA, Brazil; 5 Veterinarian, DSc., PPGV, IEC, Ananindeua, PA, Brazil; 6 Biomedical Scientist, DSc, Seção de Arbovirologia e Febres Hemorrágicas, IEC, Ananindeua, PA, Brazil; 7 Dentist, DSc., Instituto Ciências da Saúde, Universidade Federal do Pará (UFPA), Belém, PA, Brazil

**Keywords:** equids, serology, Eastern equine encephalitis virus, Alphavirus, Brazil, equídeos, sorologia, vírus da Encefalite equina leste, Alfavírus, Brasil

## Abstract

Serum samples from 89 equids were analyzed (75 horses, 9 donkeys, and 5 mules) from the municipality of Viseu, Pará state, Brazil. Samples were collected in November 2014 and August 2015. The antibody prevalence against the following alphaviruses was estimated: Eastern equine encephalitis virus, Western equine encephalitis virus, Mucambo virus, and Mayaro virus. Seroprevalence was determined by the hemagglutination inhibition (HI) technique. Sera that exhibited HI antibodies with heterotypic reactions for the analyzed viruses were subjected to the 90% plaque reduction neutralization test (PRNT_90_). The HI prevalence of monotypic reactions to EEEV was 7.9%, and that of WEEV was 1.1%, as confirmed by PRNT_90_. Viral isolation attempts were negative for all tested blood samples. Our results suggest the circulation of equine encephalitis complex viruses. Future studies should evaluate the possible involvement of arthropod hosts and residents in the viral transmission in the study area.

## Resumo

Foram analisadas amostras de soros de 89 equídeos (75 equinos, 9 asininos e 5 muares) oriundos do município de Viseu, estado do Pará. As amostras foram coletadas no período de novembro de 2014 e agosto de 2015. Foram estimadas as prevalências de anticorpos contra os seguintes Alfavírus: vírus da Encefalite equina leste (EEEV), vírus da Encefalite equina oeste (WEEV), vírus Mucambo (MUCV), and vírus Mayaro (MAYV). A soroprevalência foi determinada por técnicas de Inibição da Hemaglutinação (IH), porém os soros que apresentaram anticorpos IH com reações heterotípicas para os vírus analisados foram submetidos ao PRNT_90_. A prevalência de reações monotípicas por IH para EEEV foi de 7, 9% e para WEEV foi de 1,1%, confirmadas por PRNT_90_. A tentativa de isolamento viral foi negativa em todas as amostras de sangue testadas. Nossos resultados sugerem a circulação de vírus do complexo das encefalites equinas. Estudos futuros devem ser realizados para avaliar possível envolvimento de hospedeiros artrópodes e residentes na área de abrangência do estudo.

## Introduction

*Eastern equine encephalitis virus* (EEEV) is an arthropod-borne virus (arbovirus) belonging to the genus *Alphavirus*, family *Togaviridae* (Chen et al., 2018). EEEV was first isolated in 1933 from horse brain tissue obtained after an outbreak in New Jersey and Virginia, United States (TenBroeck & Merrill, 1933). EEEV is enzootic in North America along the Atlantic and Gulf Coasts up to Texas, the Caribbean, and Central America. EEEV are also enzootic along the north and east coasts of South America and the Amazon River basin (Causey et al., 1962). The main transmitters of EEEV in North America are mosquitoes of the genus *Culiseta* (*C. melanura*), while in South and Central America, the main transmitters are *Culex* spp. mosquitoes, mainly *Culex pedroi*. Several other mosquito species may also transmit EEEV as secondary vectors to wild birds and animals during maintenance cycles. Mammals are also susceptible, especially horses, and epizootics have been attributed to *Aedes taeniorhynchus*, which transmits the virus among horses (Causey et al., 1962; Cupp et al., 2003; Mitchell et al., 1992; Scott & Weaver 1989; Vasconcelos et al., 1991). In general, equids and humans are accidental hosts, while wild birds are the primary hosts and amplifiers of EEEV (Gibbs, 1976; Mitchell et al., 1992). Transmission is seasonal in temperate regions, peaking in late summer or early fall. In tropical and subtropical regions, such as South America, the Caribbean, and Florida, the cycle occurs year-round (Scott & Weaver, 1989).

Among the viruses of the equine encephalitis complex, EEEV has the highest virulence and pathogenicity, and it is unique in the complex that has been sporadically isolated in Brazil from horses exhibiting clinical signs of central nervous system diseases (Campos et al., 2013; Silva et al., 2011, 2017). EEEV infections are quite variable, ranging from unapparent infections to fatal neurological diseases. After an average incubation period of 3 to 14 days, fever and depression occur, which typically go unnoticed, and the animal may recover or progress to neurological disease. Depending on the viral strain, the neurological disease may be more severe, presenting the following clinical signs: motor incoordination, ataxia, hyperexcitability, photophobia, blindness, dysphagia, tooth grinding, pressing of the head against firm objects, walking in circles, progressive paralysis, coma, and death. The mortality rate in severe cases ranges from 80% to 90%. Animals that recover after a mild disease course may exhibit neurological sequelae (Barros, 2007; Flores, 2007; Scott & Weaver, 1989).

In Brazil, serological studies of EEEV have shown the presence of specific antibodies for this virus in many different animal groups, such as wild birds (Araújo et al., 2012; Dégallier et al., 1992; Ferreira et al., 1994; Shope et al., 1966), marsupials (Shope et al., 1966), and species such as sheep (*Ovis aries*) (Pauvolid-Corrêa et al., 2015), capuchin monkeys (*Cebus libidinosus*) (Laroque et al., 2014), jaguars (*Panthera onca*) (Onuma et al., 2016), and sloths (*Bradypus torquatus*) (Catenacci et al., 2018), among others.

The state of Pará has the highest deforestation levels in the Brazilian Amazon region (Prates & Serra, 2009), which increases risk of infection with arboviruses among the local population. The present study aimed to investigate the prevalence of antibodies against four arboviruses belonging to the genus *Alphavirus* (family *Togaviridae*) in equids in Viseu, a municipality in the eastern region of Pará state located at the border with the state of Maranhão, in which there have been no serological studies on *Alphavirus* circulation in equids.

## Materials and methods

The study samples were collected after written consent from the owners of the animals, in accordance with the Ethical Principles in Animal Experimentation adopted by the National Council for Animal Control and Experimentation (Conselho Nacional de Controle e Experimentação Animal-CONCEA), and approved by the Ethics Committee on the Use of Animals of the Instituto Evandro Chagas (CEUA/IEC license, under protocol no. 28/2014 and certification no. 021/2014).

**Figure 1:**
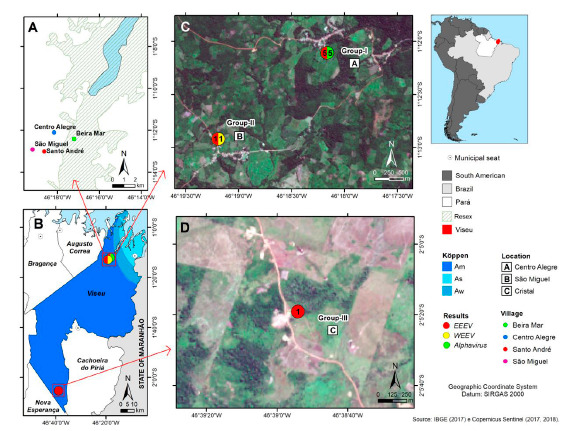
Map of the study sites in Viseu municipality, State of Pará, Brazil. (A) the sampling sites in all villages, Viseu in Para State; (B) the distance between the sampling sites; (C) and (D) positive results from the HI and PRNT_90_ for *Alphavirus* tests. The arrows point to the capture sites of the animals.

The study was conducted in the municipality of Viseu, state of Pará (40°3939″ N, 7°5434″ W), at two different time points (December 2014 and August 2015) and sites. The first study site (Figure 1A and 1C) covered four small rural locations in Açaiteua (Centro Alegre, Beira Mar, Santo André, and São Miguel), which have native and highly fragmented forest areas constituting secondary forests. The other study site was in Viseu, Mejer Farm, located in the village of Cristal, 100 km from Açaiteua (Figure 1D). The climate in the municipality of Viseu (Figure 1B) is classified as tropical, type Am, As, and Aw according to the Köppen climate classification (Alvares et al., 2013), and has a relatively high temperature (mean of 28.3°C) and rainfall levels with a humidity of 77.6%.

All healthy equids with no record of vaccination against viruses causing equine encephalitis were included in the study. These animals were classified into three groups: group I, free-ranging (where they live) animals with forest access (n = 17); group II, enclosed draft animals, only going into the forest to perform some activity (n = 18); group III, farm animals living in a semi-intensive system (n = 54).

Information was collected using an individual form, which contained the following information: owner’s name, animal name and/or number, date of travel, survey location, group, species (horse, donkey, mule), breed, sex, age (<1 year, 1 to ≤ 5 years, >5 years, not informed [NI]), utility (work, sport, riding, reproduction, other, NI), female reproductive status (pregnant or not), history of vaccination against EEEV or *Western equine encephalitis virus* (WEEV), type of biological sample collected, and laboratory results. A total of 10 mL of blood was collected from each animal by venipuncture of the jugular vein. After coagulation, the serum sample was separated into two aliquots. One was immediately stored in liquid nitrogen for viral isolation, and the other was stored at -20°C for serological tests.

Antibodies were detected by the hemagglutination inhibition (HI) test as described by Clarke & Casals (1958) and adapted to microplates by Shope (2002). Sera were tested for four arboviruses belonging to the genus *Alphavirus*: EEEV (strain BE AN7526), WEEV (strain BE AN7100), *Mayaro virus* (MAYV) (strain BE AR20290), and *Mucambo virus* (MUCV) (strain BE AN10967). Samples with titers greater than or equal to 1:20 were considered positive. The sera that presented cross-reactions (heterotypic) in the HI test were subjected to the 90% plaque reduction neutralization test (PRNT_90_) to determine which virus caused the infection. PRNT_90_ was performed according to the protocol adapted from Tauro et al. (2012), and serum samples with antibody titers greater than or equal to 1:20 were considered positive for the viruses tested. When positive for more than one of the viruses tested, the serological sample was considered positive for *Alphavirus*.

For viral isolation, 25 µL of blood diluted at 1:10 in the L-15 culture medium was inoculated onto monolayers of *Aedes albopictus* clone C6/36 cells (ATCC®) supplemented with L-15 medium plus 2.05 mM l-glutamine (Sigma®), fetal bovine serum (5%) (Invitrogen®), tryptose phosphate (2.95%), antibiotics (penicillin 10,000 U/L, streptomycin 10,000 µg/L), and non-essential amino acids (10 mL/L). Cells were maintained at 28°C (Igarashi, 1978). The culture medium was observed daily for ten days to visualize possible cytopathic effects. The presence or absence of the viral agent in the cell culture was determined by an indirect immunofluorescence test using a polyclonal antibody against alphaviruses, as described by Gubler et al. (1984).

The equid sample collection sites were georeferenced using the global positioning system (GPS) Garmin GPSMap 64s, and the data were transferred to ArcGIS 10.3 software (https://www.arcgis.com) to draw distribution maps of the arboviruses. The state and municipal boundaries were obtained from the Brazilian Institute of Geography and Statistics (IBGE) (http://www.ibge.gov.br/). The Köppen climate classification data were obtained from Alvares et al. (2013). The satellite images were generated using the Sentinel 2 sensor of the European Space Agency (ESA) (https://sentinel.esa.int/web/sentinel/user-guides/sentinel-2-msi) with the CC-BY Open Access License (http://open.esa.int/) from 2017 and 2018.

The G test was used to analyze independence between HI seroprevalence rates and neutralizing antibodies and independence between animal groups, the two sexes, and age groups. Next, an exploratory analysis was performed between the variables with significant associations in the correspondence analysis, aimed at synthesizing the variability structure of the data in terms of dimensions, in which the number of dimensions was smaller than the number of variables. A significance level of 5% was adopted for all analyses.

## Results

A total of 89 equids were sampled, including horses (*n* = 75), donkeys (*n* = 9), and mules (*n* = 5), with estimated ages of between 6 months and 20 years. There were 36 (40.45%) females and 53 (59.55%) males (Table 1, Box 2 in Supplementary Material). Viral isolation attempts were negative in all investigated samples. Of the 89 serum samples analyzed by the HI test, 13/89 (14.6%) showed heterotypic reactions (HRs) to all four viruses (EEEV, WEEV, MUCV, and MAYV), and in 6/89 (6.7%) sera, a monotypic reaction (MR) to EEEV was detected. The sera that exhibited HI antibodies with HRs to EEEV, WEEV, MAYV, and MUCV were subjected to PRNT_90_, and an MR was confirmed in two samples, one for EEEV and the other for WEEV, both from animals in group II (Table 1). Regarding the other HRs, one remained inconclusive, and the other ten samples did not replicate the HI reactivity to the *Alphavirus* species as tested by PRNT_90_, suggesting that the infection may have been caused by another untested *Alphavirus* (Tables 2 and 3 in Supplementary material).

**Table 1:** Prevalence of antibodies to *Alphavirus* detected by the hemagglutination inhibition test (HI) and 90% plaque reduction neutralization test (PRNT_90_) by animal group, sex, and age in equine serum.

Variables	HI						Total		p*
	EEEV (MR)		HR		Negative				
	n	%	n	%	n	%	n	%	
*Group*									<0.001
*G1*	5	29.4	5	29.4	7	41.2	17	100.0	
*G2*	0	0.0	4	22.2	14	77.8	18	100.0	
*G3*	1	1.9	4	7.4	49	90.7	54	100.0	
*Sex* ^*#*^									0.572
*Female*	2	5.6	7	19.4	27	75.0	36	100.0	
*Male*	4	7.7	6	11.5	42	80.8	52	100.0	
*Age* ^*¥*^									0.351
*≤ 5 years*	4	11.4	3	8.6	28	80.0	35	100.0	
*> 5 years*	2	4.2	7	14.6	39	81.2	48	100.0	
*Total*	6	6.7	13	14.6	70	78.7	89	100.0	------
Variables	PRNT						Total		p*
	EEEV		WEEV		Negative				
	n	%	n	%	n	%	n	%	
Group									0.008
*G1*	5	29.4	0	0.0	12	70.6	17	100.0	
*G2*	1	5.6	1	5.6	16	88.9	18	100.0	
*G3*	1	1.9	0	0.0	53	98.1	54	100.0	
*Sex* ^*#*^									0.324
*Female*	2	5.6	1	2.8	33	91.7	36	100.0	
*Male*	5	9.6	0	0.0	47	90.4	52	100.0	
*Age* ^*¥*^									0.707
*≤ 5 years*	4	11.4	0	0.0	31	88.6	35	100.0	
*> 5 years*	3	6.2	0	0.0	45	93.8	48	100.0	
*Total*	7	7.9	1	1.1	70	78.7	89	100.0	------

EEEV, Eastern equine encephalitis virus; WEEV, Western equine encephalitis virus; HI, Haemaglutination inhibition; PRNT_90_, 90% plaque reduction neutralization test; HR, Heterotypical reaction; N, absolute number

*G test’s *p*-value. ^#^1 animal without sex information ^¥^6 animals without age information

**Figure 2:**
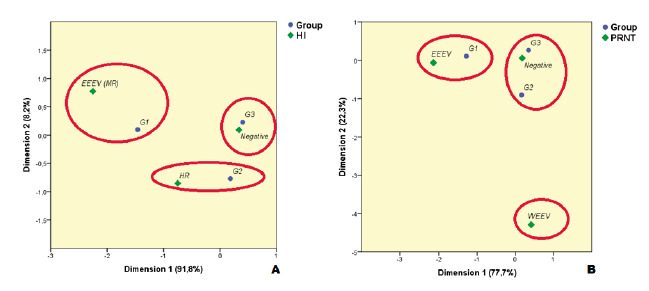
Biplots in correspondence analyses of animal groups and results of serological tests. (A) Hemagglutination Inhibition test result (HI); (B) Plaque reduction neutralization (PRNT).

According to the serological results of HI and PRNT_90_, the prevalence of MRs to EEEV was 7.9%. The prevalence was higher in group I (29.4% in both tests), establishing correlations (p < 0.05 for both) between the animal group and both serological tests (HI and PRNT_90_). The frequencies (prevalence) observed from the test results did not differ by animal sex or age (Table 1). Of the seven equids seropositive for EEEV, five were from group I, one from group II, and one from group III, with HI antibody titers of 40 (*n* = 2), 80 (*n* = 2), 160 (*n* = 2), and 360 (*n* = 1) (Figure 2).

The profile of the horse group and the results of the serological tests were significantly associated. Dimensions 1 and 2 explained 91.8% and 8.2%, respectively, of the total principal inertia (variance), with dimension 1 being the most important in the behavior of the data. In total, the correspondence analysis models explained 28.4% (in the HI × group relationship) and 19.9% (in the PRTN_90_ × group relationship) of the variation in the data (Table 2 in Supplementary material). In dimension 1, we could better observe the variation of the results in comparison between both variables (Table 3 in Supplementary material). The categories of a positive result for EEEV and animal group explained the significant variation in the associations between the tests and the animal group, forming a cluster, as shown in Figure 1. An association was also observed between a positive result for HR and group II, suggesting the circulation of alphaviruses in this area. Group III showed a frequency of 60.7% (mass = 0.607) out of the total number of animals, and horses had a higher frequency of negative results (Figure 2 and Table 3 in Supplementary material).

**Table 2:** Eigenvalues and inertia of dimensions verified in correspondence analysis of the results of serological tests associated with groups of animals.

Test	Dimension	Eigenvalue	Inertia	Inertia proportion (%)	X^2^
HI	1 (X axis)	0.511	0.261	91.8	
	2 (Y axis)	0.153	0.023	8.2	
	Total	-------	0.284	100.0	25.311*
PRNT	1 (X axis)	0.393	0.154	77.7	
	2 (Y axis)	0.210	0.044	22.3	
	Total	-------	0.199	100.0	17.674*

HI, Haemaglutination inhibition. *p<0,05.

**Table 3:** Coordinates and and contributions from categories of animal groups and results of serological tests obtained in correspondence analysis.

Variables	Geral		Dimension 1 (X axis)		Dimension 2 (Y axis)	
	Mass	% inertia	Score	CTR	Score	CTR
IH and Group						
HI						
EEEV (MR)	0.067	0.181	-2.252	0.669	0.773	0.264
HR	0.146	0.058	-0.750	0.161	-0.852	0.693
Negative	0.787	0.045	0.332	0.170	0.092	0.043
Group						
1	0.191	0.208	-1.460	0.797	-0.097	0.012
2	0.202	0.022	0.180	0.013	0.770	0.785
3	0.607	0.054	0.400	0.190	-0.226	0.203
Total	1.000	0.284	-------	1.000	-------	1.000
PRTN and Group						
PRTN						
WEEV	0.011	0.044	0.415	0.005	-4.292	0.984
EEEV	0.079	0.142	-2.143	0.920	-0.062	0.001
Negative	0.910	0.012	0.180	0.075	0.058	0.015
Group						
1	0.191	0.124	-1.281	0.798	0.109	0.011
2	0.202	0.037	0.163	0.014	-0.903	0.784
3	0.607	0.038	0.349	0.188	0.267	0.205
Total	1.000	0.199	-------	1.000	-------	1.000

CTR: contribution of the profile to the inertia of the axis; HI: Haemaglutinatio inhibition.

## Discussion

This is the first report on the circulation of antibodies against members of the genus *Alphavirus* in equids raised on farms in the eastern Brazilian Amazon. We detected the prevalence of HI antibodies to four *Alphavirus* species, which provided strong serological evidence of EEEV and WEEV circulation in the study area.

Statistical analysis revealed significant results when comparing groups I and II, indicating that only these horses showed serological reactivity. Regarding the presence of HI antibodies, the prevalence of MRs for EEEV was significantly higher in group I than in groups II and III. The HR for alphaviruses was associated with group II, indicating the active circulation of members of the genus *Alphavirus* in the region. The association of group I with EEEV in PRNT_90_ reinforces evidence of recent EEEV infection in the study areas. The association of groups II and III with negative PRNT_90_ results, despite HI positivity, reinforces the possibility of previous infection of these animals by alphaviruses before the study period. Therefore, we suggest that in group I, since the horses lived close to the edge of the forest and were prone to direct contact with wild animals and potential vectors, they may have been infected but remained asymptomatic. The detection of antibodies to EEEV by PRNT_90_ indicates either a distant past or very recent exposure to EEEV. It is important to mention that in the 1960s, an epizootic caused by EEEV was diagnosed in the municipality of Bragança in the vicinity of Viseu (Causey et al., 1962).

Reactivity to EEEV increased with age, occurring at greater percentages in animals older than five years, in line with the observation reported by Pauvolid-Corrêa et al. (2015), who showed that seropositivity to EEEV increased with age in samples of equids from the Nhecolândia subregion of the Brazilian Pantanal in the middle-western region.

The present study detected 7.9% reactive animals, corroborating studies conducted to detect arboviruses in the state of Pará by Heinemann et al. (2006) and Casseb et al. (2016), who found serological evidence of EEEV in equids, with prevalence of antibodies against EEEV of 27.37 and 9.82%, respectively. Together, these studies suggest that EEEV circulation has been active for nearly two decades or longer in the state. All seroreactive animals showing a monotypic response to EEEV had no history of travel to other locations or vaccination, implying that transmission may have occurred in the region studied.

Previous serological surveys reported the prevalence of antibodies against EEEV in the Brazilian Amazon and other Brazilian states, including studies by Aguiar et al. (2008) in the state of Rondônia (21% prevalence); Cunha et al. (2009) in the state of São Paulo (16% prevalence); Pauvolid-Corrêa et al. (2010) in the South Pantanal (47.7% prevalence); Melo et al. (2012) in the Amazon, Pantanal, and Cerrado biomes of the state of Mato Grosso (35.5% prevalence); Lara et al. (2014) in the state of Minas Gerais (30.2% prevalence); and Pauvolid-Corrêa et al. (2015) in the state of Mato Grosso do Sul (18% prevalence).

Another outbreak caused by EEEV has been reported by Silva et al. (2011) in the states of Pernambuco, Ceará, and Paraíba, who found 100% of the tested animals infected with EEEV by real-time PCR and sequencing. Epizootics have also been reported in horses in the state of Paraíba by Araújo et al. (2012), who reported variable prevalence rates of EEEV in animals with clinical symptoms (12.8%), 54.3% by the HI test, and 63.7% by PRNT_90_. A study by Campos et al. (2013) identified EEEV in two of the three horses tested from Marajó Island, Pará state, by semi-nested real-time PCR. Together with ours, these findings emphasize that EEEV circulation in equids in Brazil is active and, therefore, widely distributed in the Brazilian territory.

None of the four equine encephalitis complex viruses included in this study were detected in the 89 analyzed biological samples, indicating that the equids did not carry the virus at the time of blood collection. Nonetheless, this finding may also be due to the low sampling of animals, especially in area I, which showed a higher prevalence of positive serology for EEEV due to its proximity to the forest area. In addition, the surveyed animals in the study were asymptomatic and healthy. According to Pauvolid-Corrêa et al. (2014), equids are often used as indicators of arbovirus circulation in a given location because they are often exposed to mosquito vectors. However, Gibbs (1976) argues that the viremia level of EEEV in horses is low.

## Conclusions

Encephalitis is one of the most serious clinical manifestations caused by arboviruses. It can lead to death or serious sequelae in humans and surviving animals, making it an important public and veterinary health problem. The results obtained in this study suggest the existence of a transmission cycle of the alphaviruses studied at the research location associated with the Amazon biome’s tropical climate. We also confirmed the circulation of EEEV in equine populations in the state of Pará, which has a favorable environment for the maintenance of EEEV in nature, with the presence of vectors and extensive bird biodiversity. However, there are no confirmed cases of severe illness in humans, and the epidemics detected have been rare.

On the other hand, the lack of recent data related to the circulation of EEEV in the state of Pará, associated with reports of symptomatic infection in horses in the country in the last decades, shows the need to implement effective epidemiological surveillance of human and veterinary health with continuous monitoring of the circulation of these agents in the Amazon and other Brazilian biomes.

## Supplementary Material

Supplementary material accompanies this paper.

Box 2 Information from equines participating in the study

This material is available as part of the online article from http://rbmv.org/index.php/BJVM.
