# Supplementary material for: Serological evidence of Eastern equine encephalitis circulation in equids in Pará state, Brazil
Source: Braz J Vet Med. 2021 Mar 31;43:e001720. doi: 10.29374/2527-2179.bjvm001720 (PMC9185993; doi:10.29374/2527-2179.bjvm001720)
Supplement: Supplementary file 1 [file bjvm-43-e001720-suppl1.pdf]

Supplementary Material

Box 2. Information from equines participating in the study

CODE	DATE SAMPLES	SITE	GROUP	SPECIE	GENDER	AGE
BeAN 808911	Nov/2014	C.A	I	Equine	F	> 5
BeAN 808913	Nov/2014	C.A	I	Equine	F	1 a ≤ 5
BeAN 808915	Nov/2014	C.A	I	Equine	F	> 5
BeAN 808917	Nov/2014	C.A	I	Equine	M	<1
BeAN 808919	Nov/2014	C.A	I	Equine	M	1 a ≤ 5
BeAN 808921	Nov/2014	C.A	I	Equine	M	1 a ≤ 5
BeAN 808923	Nov/2014	C.A	I	Equine	F	1 a ≤ 5
BeAN 808925	Nov/2014	C.A	I	Equine	F	<1
BeAN 822710	Aug/2015	C.A	I	Equine	F	1 a ≤ 5
BeAN 822711	Aug/2015	C.A	I	Equine	M	1 a ≤ 5
BeAN 822712	Aug/2015	C.A	I	Equine	F	> 5
BeAN 822713	Aug/2015	C.A	I	Equine	F	<1
BeAN 822714	Aug/2015	C.A	I	Equine	F	> 5
BeAN 822715	Aug/2015	C.A	I	Equine	F	1 a ≤ 5
BeAN 822716	Aug/2015	C.A	I	Equine	M	<1
BeAN 822717	Aug/2015	C.A	I	Equine	M	<1
BeAN 822718	Aug/2015	C.A	I	Equine	F	<1
BeAN 822719	Aug/2015	B.M	II	Donkey	M	1 a ≤ 5
BeAN 822720	Aug/2015	B.M	II	Donkey	M	<1
BeAN 822721	Aug/2015	B.M	II	Donkey	F	> 5
BeAN 822722	Aug/2015	S.A	II	Donkey	M	> 5
BeAN 822723	Aug/2015	S.A	II	Equine	M	UI
BeAN 822724	Aug/2015	S.A	II	Donkey	M	> 5
BeAN 822725	Aug/2015	S.A	II	Donkey	M	> 5
BeAN 822726	Aug/2015	S.A	II	Equine	M	> 5
BeAN 822727	Aug/2015	S.A	II	Burro	M	> 5
BeAN 822728	Aug/2015	S.M	II	Donkey	F	UI

BeAN 822729	Aug/2015	S.M	II	Donkey	M	UI
BeAN 822730	Aug/2015	S.M	II	Donkey	F	UI
BeAN 822731	Aug/2015	S.M	II	Donkey	M	> 5
BeAN 822732	Aug/2015	S.M	II	Donkey	M	> 5
BeAN 822733	Aug/2015	S.M	II	Mule	F	> 5
BeAN 822734	Aug/2015	S.M	II	Mule	F	UI
BeAN 822735	Aug/2015	S.M	II	Equine	M	UI
BeAN 822736	Aug/2015	S.M	II	Equine	M	> 5
BeAN 822737	Aug/2015	C	III	Equine	F	> 5
BeAN 822738	Aug/2015	C	III	Equine	F	> 5
BeAN 822739	Aug/2015	C	III	Equine	F	> 5
BeAN 822740	Aug/2015	C	III	Equine	F	> 5
BeAN 822741	Aug/2015	C	III	Equine	F	> 5
BeAN 822742	Aug/2015	C	III	Equine	F	> 5
BeAN 822743	Aug/2015	C	III	Equine	F	> 5
BeAN 822744	Aug/2015	C	III	Equine	F	> 5
BeAN 822745	Aug/2015	C	III	Equine	F	1 a ≤ 5
BeAN 822746	Aug/2015	C	III	Equine	F	> 5
BeAN 822747	Aug/2015	C	III	Equine	F	> 5
BeAN 822748	Aug/2015	C	III	Equine	F	> 5
BeAN 822749	Aug/2015	C	III	Equine	F	> 5
BeAN 822750	Aug/2015	C	III	Equine	F	> 5
BeAN 822751	Aug/2015	C	III	Equine	F	> 5
BeAN 822752	Aug/2015	C	III	Equine	F	> 5
BeAN 822753	Aug/2015	C	III	Equine	F	> 5
BeAN 822754	Aug/2015	C	III	Equine	F	> 5
BeAN 822755	Aug/2015	C	III	Equine	F	1 a ≤ 5
BeAN 822756	Aug/2015	C	III	Equine	F	> 5
BeAN 822757	Aug/2015	C	III	Equine	M	1 a ≤ 5
BeAN 822758	Aug/2015	C	III	Equine	M	> 5

BeAN 822759	Aug/2015	C	III	Equine	M	1 a ≤ 5
BeAN 822760	Aug/2015	C	III	Equine	M	1 a ≤ 5
BeAN 822761	Aug/2015	C	III	Equine	M	> 5
BeAN 822762	Aug/2015	C	III	Equine	M	> 5
BeAN 822763	Aug/2015	C	III	Equine	M	1 a ≤ 5
BeAN 822764	Aug/2015	C	III	Equine	M	<1
BeAN 822765	Aug/2015	C	III	Equine	M	> 5
BeAN 822766	Aug/2015	C	III	Equine	M	> 5
BeAN 822767	Aug/2015	C	III	Equine	M	<1
BeAN 822768	Aug/2015	C	III	Equine	M	> 5
BeAN 822769	Aug/2015	C	III	Equine	M	> 5
BeAN 822770	Aug/2015	C	III	Equine	M	> 5
BeAN 822771	Aug/2015	C	III	Equine	M	<1
BeAN 822772	Aug/2015	C	III	Equine	M	> 5
BeAN 822773	Aug/2015	C	III	Equine	M	1 a ≤ 5
BeAN 822774	Aug/2015	C	III	Equine	M	1 a ≤ 5
BeAN 822775	Aug/2015	C	III	Equine	M	> 5
BeAN 822776	Aug/2015	C	III	Equine	M	1 a ≤ 5
BeAN 822777	Aug/2015	C	III	Equine	M	1 a ≤ 5
BeAN 822778	Aug/2015	C	III	Equine	M	1 a ≤ 5
BeAN 822779	Aug/2015	C	III	Equine	M	> 5
BeAN 822780	Aug/2015	C	III	Equine	M	1 a ≤ 5
BeAN 822781	Aug/2015	C	III	Equine	M	1 a ≤ 5
BeAN 822782	Aug/2015	C	III	Equine	M	1 a ≤ 5
BeAN 822783	Aug/2015	C	III	Equine	M	1 a ≤ 5
BeAN 822784	Aug/2015	C	III	Equine	M	> 5
BeAN 822785	Aug/2015	C	III	Equine	M	> 5
BeAN 822786	Aug/2015	C	III	Equine	M	> 5
BeAN 822787	Aug/2015	C	III	Equine	M	1 a ≤ 5
BeAN 822788	Aug/2015	C	III	Equine	M	> 5

BeAN 822789	Aug/2015	C	III	Equine	M	<1
BeAN 822790	Aug/2015	C	III	Equine	M	> 5
